# Unpacking the intention to action gap: a qualitative study understanding how physicians engage with audit and feedback

**DOI:** 10.1186/s13012-021-01088-1

**Published:** 2021-02-17

**Authors:** Laura Desveaux, Noah Michael Ivers, Kim Devotta, Noor Ramji, Karen Weyman, Tara Kiran

**Affiliations:** 1 Women’s College Hospital Institute for Health Systems Solutions and Virtual Care, 76 Grenville Ave Toronto, Toronto, Ontario Canada; 2 Institute for Health Policy, Management & Evaluation, University of Toronto, 155 College St, Toronto, Ontario Canada; 3 Department of Family and Community Medicine, University of Toronto, 500 University Avenue, Toronto, Ontario Canada; 4 MAP Centre for Urban Health Solutions, Li Ka Shing Knowledge Institute of St. Michael’s Hospital, 30 Bond Street, Toronto, Ontario Canada; 5 Dalla Lana School of Public Health, University of Toronto, 155 College St, Toronto, Ontario Canada; 6 Department of Family and Community Medicine, St. Michael’s Hospital, 30 Bond Street, Toronto, Ontario Canada

**Keywords:** Audit and feedback, Primary care, Practice improvement, Physician learning, Implementation

## Abstract

**Background:**

Audit and feedback (A&F) often successfully enhances health professionals’ intentions to improve quality of care but does not consistently lead to practice changes. Recipients often cite data credibility and limited resources as barriers impeding their ability to act upon A&F, suggesting the intention-to-action gap manifests while recipients are interacting with their data. While attention has been paid to the role feedback and contextual variables play in contributing to (or impeding) success, we lack a nuanced understanding of how healthcare professionals interact with and process clinical performance data.

**Methods:**

We used qualitative, semi-structured interviews guided by Normalization Process Theory (NPT). Questions explored the role of data in quality improvement, experiences with the A&F report, perceptions of the data, and interpretations and reflections. Interviews were audio-recorded and transcribed verbatim. Data were analyzed using a combination of inductive and deductive strategies using reflexive thematic analysis informed by a constructivist paradigm.

**Results:**

Healthcare professional characteristics (individual quality improvement capabilities and beliefs about data) seem to influence engagement with A&F to a greater degree than feedback variables (i.e., delivered by peers) and observed contextual factors (i.e., strong quality improvement culture). Most participants lacked the capabilities to interpret practice-level data in an actionable way despite a motivation to engage meaningfully. Reasons for the intention-to-action gap included challenges interpreting longitudinal data, appreciating the nuances of common data sources, understanding how aggregate data provides insights into individualized care, and identifying practice-level actions to improve quality. These factors limited effective cognitive participation and collective action, as outlined in NPT.

**Conclusions:**

A well-designed A&F intervention is necessary but not sufficient to inform practice changes. A&F initiatives must include co-interventions to address recipient characteristics (i.e., beliefs and capabilities) and context to optimize impact. Effective strategies to overcome the intention-to-action gap may include modelling how to use A&F to inform practice change, providing opportunities for social interaction relating to the A&F, and circulating examples of effective actions taken in response to A&F. More broadly, undergraduate medical education and post-graduate training must ensure physicians are equipped with QI capabilities, with an emphasis on the skills required to interpret and act on practice-level data.

**Supplementary Information:**

The online version contains supplementary material available at 10.1186/s13012-021-01088-1.

Contributions to the literature

- Research has identified that feedback variables, contextual variables, and recipient variables influence feedback, but little is known about whether and how these variables interact to influence the recipient’s ability to act on the data
- Recipient variables, including individual capabilities and beliefs about data, influence engagement with A&F to a greater degree than feedback variables (i.e., delivered by peers) and observed contextual factors (i.e., a strong quality improvement culture)
- Co-interventions including peer support and training to improve quality improvement skills may be helpful to overcome the intention-to-action gap and enable systematic changes in clinical practice.

## Background

The potential for audit and feedback (A&F) to improve quality of care is well-established [1], with recent implementation research focusing on identifying strategies to enhance its effectiveness [2, 3]. While A&F influences recipients’ intentions to improve quality of care [4], health professionals consistently report not acting on their data [5]. A recent qualitative synthesis of 65 studies developed a healthcare-specific theory of A&F, Clinical Performance Feedback Intervention Theory (CP-FIT), which highlighted three types of variables that operate through a set of common explanatory mechanisms to influence whether and how health professionals respond to A&F: feedback variables, contextual variables, and recipient variables [6]. While CP-FIT describes variables and mechanisms for A&F to successfully lead to improvements in patient care, gaps remain in our understanding of how to effectively influence them.

Several studies have explored recipient *reactions* to A&F to address this gap, with oft-cited barriers relating to data credibility and limited resources (explanatory mechanisms that predict success, or lack thereof) [5–7]. This suggests the intention-to-action gap likely manifests while recipients are interacting with their data. While much attention has been paid to the role feedback and contextual variables play in contributing to (or impeding) success, we lack a nuanced understanding of how recipients interact with and process data. For example, feedback self-efficacy (an individual’s ability to interpret and respond to feedback appropriately) is a predictor of participation in professional development activities [8], but how this relates to the intention-to-action gap in the context of A&F has not been explored. Understanding how recipients’ interact with and process their data to form (or fail to form) their behavioural response is central to the ability to effectively support them in addressing the intention-to-action gap.

Normalization Process Theory (NPT) [9] offers a theory-based lens through which to explore this gap. NPT is a sociological theory used to understand the implementation, embedding, and integration of new approaches in healthcare settings [10]. It accounts for how people understand and make sense of an approach (coherence), engage and participate with it (cognitive participation), distribute work (collective action), and reflect or appraise its effects (reflexive monitoring) [9]. Despite the conceptual similarities between these constructs and stages of CP-FIT, prior A&F research has not utilized NPT as the lens through which to describe how A&F does (or does not) influence positive practice change. NPT provides a framework for understanding whether, how, and why health professionals normalize the use of A&F to make practice changes—a necessary step for A&F to impact quality of care. To that end, the current study aimed to understand how primary care physicians interact with A&F. Specifically, the objectives were to (1) understand how physicians cognitively engage with A&F and (2) explore ways to close the intention-to-action gap.

## Methods

### Study design

We used a qualitative approach to understand how physicians engage with their data and the factors that influence engagement. The protocol received ethics approval from the St. Michael’s Hospital Research Ethics Board.

### Context and setting

Family Health Teams are publicly funded primary care organizations in Ontario, Canada, which include an interprofessional team (e.g., physicians, nurse practitioners, registered nurses, social workers, etc.) who work together to provide care for individuals in their community [11]. The St. Michael’s Hospital Academic Family Health Team (SMHAFHT) is a large primary care organization with six clinics located in downtown Toronto. The team has expanded over the last decade and now includes approximately 48,000 enrolled patients, served by more than 130 health professionals, including 80 full-time and part-time physicians. Each physician in the SMHAFHT has a “roster” of patients in their own practice and has access to an interprofessional team to support care for that roster. SMHAFHT serves a diverse patient population ranging from double-income professionals to new immigrants, people living in poverty, people experiencing homelessness, and other patient populations who have traditionally faced barriers to care such as those with schizophrenia, bipolar, addictions, and HIV.

SMHAFHT has developed a strong quality improvement (QI) program that includes an interprofessional steering committee, local QI teams at each of the six clinics, and paid QI physician leads [12]. The team tracks quality of care using a dashboard of over 25 quality measures reported at the team-level and prioritizes specific areas for improvement through development of an annual QI plan. The team has successfully led improvements in after-hours access [13], cancer screening [14], and high-risk opioid prescribing [15].

### Intervention

In May 2018, all staff physicians (*n* = 73) within the SMHAFHT with an established roster of patients were provided with an individualized, confidential practice report and associated self-reflection guide (together comprising the intervention) in two ways: an electronic copy sent by email and a paper copy delivered to their mailbox. The report was developed and refined by the QI program leadership (who were staff physicians themselves), with the input of SMHAFHT physician colleagues, over a 2-year period. Feedback was solicited in a range of ways, including an electronic survey, group discussions at a faculty development workshop, and ongoing discussions at general staff meetings. The first iteration of the report was provided confidentially to physicians in November 2017 with plans to distribute regularly. The report was intended to summarize physician-level data from a range of sources to provide physicians with a comprehensive view of the demographics and quality of care for their rostered patients in a way that complemented existing team-level reports prepared semi-annually. During development of the report, QI leadership was explicit that the report was to support reflection, professional development, and practice improvement and would not be used for external evaluation, reward, or punishment. SMHAFHT physicians informed what indicators were included in the report and how it was distributed; they recommended different ways in which they could be supported to make change based on the data. In response to physician recommendations, the QI leadership developed a plan to test a series of supports sequentially, each with increasing levels of social interaction. Structured self-reflection was the first type of learning support offered with the report.

The report leveraged data from the electronic medical record, provincial reports, manual audit, and a practice patient experience survey. All physicians within the SMHAFHT were provided with aggregate and practice-level as well as clinic and provincial level comparisons when available (see Additional file 1 for an example report). Quality indicators included data on access and continuity, high-risk prescribing, prevention, and chronic disease management. Multiple indicators were included to provide physicians with the ability to select areas where the data showed room for improvement and were a priority for them personally. The structured self-reflection guide that was designed to support physicians to reflect on areas of success and areas of improvement, for both their personal practice and their clinic (see Additional file 2 for self-reflection guide). Physicians who completed the self-reflection guide could submit it to the study team for continuing medical education credits (in Canada, physicians need to submit 25 credits annually to maintain family medicine certification). Physicians received reminders via email and in-person team meetings to complete the self-reflection guide.

### Participant recruitment

Staff physicians were informed of the study by a research team member (TK) at a staff meeting in May 2018 and reminded using follow-up emails. Physicians who completed and submitted the structured self-reflection guide were offered the opportunity to participate in a semi-structured interview at their convenience, either in-person or by telephone. Interviews were conducted between June and September 2018 and recruitment continued until no new insights emerged.

### Data collection

Qualitative semi-structured interviews were conducted by a research coordinator (KD) with no clinical affiliation or existing relationship with eligible participants. The interview guide was informed by NPT [9, 16], which focuses on the work required by individuals to normalize an intervention (i.e., make it a routine part of workflows) and identifies factors that either promote or inhibit the routine incorporation of complex interventions into everyday practice. Key constructs as they relate to A&F are defined as follows: *coherence* refers to the sense-making work done to define and organize the use of A&F to inform practice change; *cognitive participation* refers to the means by which individuals participate in using A&F to think about practice change; *collective action* refers to the work undertaken to re-shape actions related to practice; and *reflexive monitoring* refers to the work done to define and organize the knowledge upon which appraisal is founded (i.e., delivering the A&F) [9]. The interview guide was pilot tested on physician members of the research team and included questions about the role of data in QI, participants’ experiences with the A&F report, their perceptions of the data, and their interpretations and reflections (Additional file 3). All interviews were audio recorded and transcribed verbatim.

Qualitative and quantitative data from the self-reflection guides were collected using MS Excel and used to inform the design of future feedback and supports. For this study, we analyzed qualitative responses to the following two questions: “Please comment on what can be done to make this data and feedback process more useful for you” and “Reflecting on the data, describe a goal for your own learning and professional development.”

### Data analysis

Qualitative interview data were first analyzed inductively using reflexive thematic analysis [17] informed by a constructivist paradigm [18]. Once inductive codes were developed, they were deductively categorized according to NPT constructs, where applicable. Two coders (KD and LD) independently read and re-read the first three transcripts to achieve immersion and familiarization with the data. These transcripts were then coded independently, and codes and associated sections of the text were compared to reflexively evaluate interpretations and to mitigate the influence of individual bias. Qualitative data from the self-reflection guides were analyzed using a content analysis to categorize responses. These data were reported as counts as the format did not allow for deeper exploration or follow-up (as in the interviews) and are therefore more reflective of the primary concerns of participants at the time of completion. The research team then met to discuss the codes and explore potential themes. Once clarity was established, one team member (KD) coded the remaining transcripts and met with a second team member (LD) regularly to review and discuss the findings. Concurrent data collection and data analysis processes allowed for interview questions to be revised in order to further explore emerging insights [19]. Codes and associated narratives were organized in NVivo and were systematically synthesized to develop the central themes. A meeting was then held with members of the broader research team (TK and NI) inviting them to challenge the themes and pose alternative interpretations. Following this meeting, potential alternate interpretations were explored, qualitative data was reviewed to ensure accuracy of the codes, and themes were revised. The team then met again to review and further refine the themes collectively.

## Results

### Semi-structured interviews

Of the 30 physicians (41%) who participated in the structured self-reflection, 23 were invited for, and 14 (61%) completed a qualitative interview (see Table 1). Four participants declined stating they were too busy while five never responded to the recruitment request. Of the seven physicians not invited for an interview, three were members of the study team and four were demographically similar to participants who had already completed interviews once saturation was achieved. Three key themes emerged that represent how participants approaching engaging with the A&F intervention. Participant responses to the self-reflection questions are categorized in Table 1 and described below.

**Table 1:** Demographic characteristics for interview participants

Study ID	Gender	Clinic site	Years in practice
ID 01	Female	Site A	11–20
ID 02	Male	Site A	0–10
ID03	Female	Site A	21+
ID04	Male	Site A	21+
ID05	Male	Site B	21+
ID06	Female	Site B	11–20
ID07	Male	Site C	11–20
ID08	Female	Site C	11–20
ID09	Male	Site C	11–20
ID10	Female	Site D	11–20
ID11	Female	Site D	21+
ID12	Male	Site E	11–20
ID13	Female	Site E	0–10
ID14	Female	Site E	11–20

### Theme 1: Perceptions of the data were the primary driver of engagement with reflexive monitoring (or lack thereof)

Participants described a range of reactions to receiving practice level data. Most participants felt that the data inadequately reflected the complexities of patient care, including the element of patient choice. Acknowledging these limitations, participants still reported the utility of the data and fell into one of two groups. They reported that the data could provide valuable insights that could drive positive changes in practice or that the data could be used as a mechanism to monitor changes in practice that were driven by other insights. Physicians in both these groups were keen to engage with the data and explore opportunities for routine use (thereby engaging in the process of reflexive monitoring).

I’m not sure that I found the area of the clinic level as helpful to me in terms of figuring out what to do with the information and figuring out where I can potentially make improvements but certainly the personal, the reflection on personal data was useful. ID06

I mean I understand why those [indicators] were chosen because that's where the data is available. I mean, it's difficult to measure healthcare and I don't know that they are the most important indicators but they are useful to have. ID12

A small minority of participants held the belief that the data was nice to know but was not useful for informing change because it did not reflect their perceived differences of their practice and/or did not align with their priorities. These individuals did not see the potential for using the data to inform QI efforts; therefore, they elected not to engage in reflexive monitoring.

I think at a minimum it tells me that I somehow am managing more patients per my clinical FTE than others. That, in addition to some of my administrative responsibilities makes me think that I'm managing more, I mean I'm not going to extrapolate much more than saying I'm managing more than maybe some of my colleagues. ID04

[Third next available appointment] I think is a very problematic measure in our department for a lot of reasons. I don't think we should stop measuring it but I have said for many years until you tell me what my continuity of care is, until you tell me what my patient satisfaction is, it doesn't matter. ID14

Participants’ perception of data accuracy led them to report it did not inform their reflections about their practice, which was further reflected in responses to the self-reflection guide. When responding to the question “Please comment on what can be done to make this data and feedback process more useful for you” (see Additional file 4), seven physicians suggested a need to improve the accuracy of the data while five provided general feedback to improve the report.

### Theme 2: Physicians struggled to interpret how aggregate practice-level data reflected their individual actions

When indicating what would make the process more useful for them, 5 of 30 physicians who completed the self-reflection guide reported a need to improve interpretation ability (e.g., the report should summarize three areas where the recipient is doing well and three areas where they can improve as they had difficulty answering this question). In the absence of data that summarized individual actions as they relate to specific patients, participants struggled to understand how aggregate (practice-level) data provided insights into individual practice patterns. A tension emerged between the relationship-based, generalist nature of primary care practice and the summative nature of practice-level feedback. Some felt that data did not accurately capture the nuances of relationship-based care or account for patient choice while others felt that the heterogeneity of primary care was not fairly reflected in a series of standalone indicators.

I think physicians naturally are socialized to focus on the individual patient centered factors and it is frustrating to that goal to have everything reduced to numbers. ID14

I think that’s one of the things that’s lacking from this is you know it doesn’t show people that were offered things like your flu vaccines and your pneumonia vaccines so we don’t get that so I think that’s a limitation. It doesn’t capture what you’re trying to do as a provider. It only captures what the patient decides to follow through on. ID06

This disconnect made it challenging for participants to draw insights from routinely measured data at the practice-level (e.g., the number of vaccinated patients) and their primary action, which they viewed in this case as offering the vaccine. This made it challenging for them to imagine how specific patients contributed to each indicator (representing an obstacle to cognitive participation), impeding their ability to generate actionable insights. Some physicians were not deterred, expressing a desire to explore opportunities for further engagement with their data by speaking with a colleague or a similarly trusted source.

I would actually need to have somebody go through this with me like a peer that I trusted with the same kind of practice as me to sort of say like well this is what I can see as a trend because I'm not sure where to go with this and I have had it sitting there on my desk for two months. ID06

### Theme 3: Physicians expressed a need for support to identify modifiable actions that drive performance

Physicians who engaged with the report described initially using the data to evaluate whether they were performing better than, similar to, or worse than their peers. Once they had completed this step, physicians struggled to understand *what* was driving their performance and *why*. While attempting to make sense of the data, most physicians suggested that several of the indicators reflected outcomes that relied on patient choice and were therefore beyond their control.

Most self-reflection responses (*n*=22/30) outlined a goal related to practice change (i.e., offer pneumovax and mammography more consistently). Despite this, interview participants described challenges specifying an action to help achieve their goal(s). When indicating what would make the process more useful for them, nine expressed a desire for support to assist with interpretation (i.e., facilitated discussion with a colleague).

I would be interested in meeting with colleagues in a group to discuss strategies- but not to tackle everything at once, but deal with one part of the data at a time. ID03

The need for support was related to a more deep-seated tension between the desire to provide high-quality patient care alongside system-level pressures, including an ever-increasing number of administrative tasks and numerous QI initiatives. Although physicians described being driven to improve their performance in theory, many were struggling with how to find the time to focus on improvement while simultaneously avoiding burnout. Participant narratives revealed that this tension was exacerbated by a pre-existing apprehension and uncertainty around a broader, system-level movement towards accountability. Participants who were anxious about data being used for accountability were initially reticent to trust the data, an emotional response that needed to be addressed to support meaningful action.

It’s not like [accountability is] a horrible thing but it’s a threatening thing. I think people see it as […] people feel like they’re working their ass off, and then somebody’s sending them back something about how you’re doing. Do you know what I mean? And it’s totally appropriate but I think it’s that emotional response. ID11

## Discussion

**Fig. 1:**
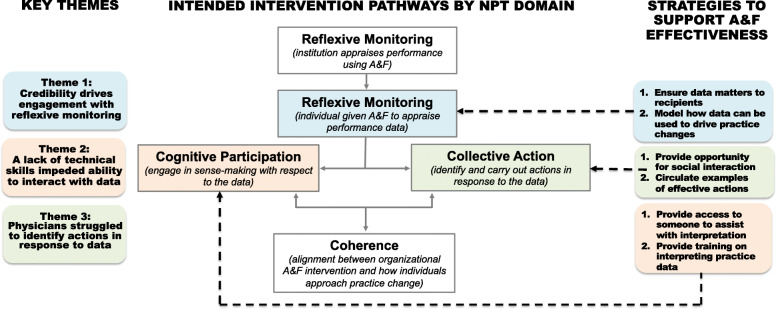
Results summary as it relates to the NPT domains. NPT = Normalization Process Theory

Our findings describe the process through which physicians engaged with an A&F intervention deployed as part of routine practice that was designed to inform practice change (see Fig. 1). Results emphasize that recipient characteristics, including individual capabilities and beliefs about data, influence engagement with A&F to a greater degree than feedback variables (i.e., delivered by peers) and observed contextual factors (i.e., a strong QI culture). Despite a motivation to engage meaningfully, participants lacked the capabilities to interpret practice-level data in an actionable way, suggesting that inaction is a product of more than just insufficient QI infrastructure [5]. This builds on our understanding of the conditions required to successfully implement A&F as part of routine practice, specifically that a strong QI culture and co-design feedback are insufficient conditions to promote practice change when recipient characteristics impede meaningful engagement. Participant recommendations suggest that modelling how to use A&F to inform practice change, providing opportunities for social interaction relating to the A&F, and circulating examples of effective actions taken in response to A&F would be useful strategies to embed alongside existing initiatives.

We found that individual beliefs and QI capabilities (or lack thereof) influenced the Interaction, Perception, and Acceptance stages outlined in CP-FIT [6], suggesting that co-interventions designed to influence views about data and build QI skills among A&F recipients are necessary and promising strategies to optimize impact. Our results suggest that relevant skills include interpreting longitudinal data, understanding common data sources and limitations and how aggregate data provides insights into individualized care, and identifying practice-level actions to improve quality. Responses to the self-reflection guide reveal that reflection questions functioned as a prompt, suggesting that reflection alone is insufficient to overcome common challenges. This further highlights a need to build QI capacity, whereby A&F recipients have the QI skills required to interpret data and the ability to identify actions in response, within an environment that supports and normalizes continuous practice improvement. This vision of QI capacity is likely to support effective *cognitive participation* as well as the transition from intention-to-action with a specific focus on the technical ability to interpret performance feedback among physician recipients of A&F [20].

Despite the ubiquitous nature of A&F, little progress has been achieved over the last decade in improving its effectiveness [5]. Physicians who receive practice data experience challenges moving beyond the notion of providing care one person at a time towards improving care for a population of patients [5]. Medical curricula and continuing professional development must provide physicians with opportunities to build the skills required to address patient and population health needs [21]. This will require a paradigm shift from the traditional notion of *QI capacity building*, that is having the right number and level of people actively engaged, to *QI capability building*, ensuring that physicians have the confidence, knowledge, and skills to improve [22].

Relatedly, the perceptions of the value of QI are inconsistent from one physician to the next, ranging from the belief that it is meaningful and important to the belief that it decreases efficiency and distracts from patient care [20]. Previous work has documented responses to A&F that primarily reflect organizational perceptions of data [7], with work at the individual level revealing negative perceptions that interfere with engagement in reflexive monitoring (data is not accurate and is therefore discounted) [23]. In contrast, many physicians in our study reported that data was imperfect but still accepted it as useful for driving meaningful change in practice. It is important to note that the feedback in this setting was physician-led with an explicit goal of learning, which may have contributed to a greater level of engagement or openness to feedback [6, 24]. Despite this approach, the way in which data was presented did not align with how participants approached practice improvement and some participants did not see the utility of the data. This suggests a need to consider existing approaches to improvement when designing A&F interventions (ideally by designing alongside recipients) and more clearly illustrate how performance feedback can be utilized and the opportunity it presents for system or clinic-level changes. Others understood the potential value of the data but were not interested in the specific indicators, highlighting the need to ensure alignment between the data contained in A&F and recipient goals [25].

The emotional tensions acting on primary care providers are a critical contextual variable precluding effective independent engagement. Although we know feedback can generate a great deal of emotion [26], the driving force behind the emotional experience is poorly understood. Our results build on prior work outlining the discordance between patient-centered ideals and system-level QI initiatives [27] by describing the nuances underlying the emotional tension surrounding physician engagement with A&F. Apprehension seems to be driven by a combination of system mistrust and the culture of autonomous practice [27]. This apprehension was present even in the context of a physician-led audit and feedback initiative. A failure to address this emotional tension coupled with the mounting expectations placed on primary care providers [28] may explain why many A&F initiatives report little to no effect. Participant feedback suggests that peer support may be a feasible option that provides additional support to enable more meaningful engagement with feedback [6] while simultaneously providing a collegial relationship to diffuse these tensions.

The goal of this qualitative study was not to generalize but rather to explore physician perspectives of an A&F intervention and provide an in-depth understanding of how and why they engaged (or failed to engage) with their personal practice data. Participants in the current study voluntarily engaged with the intervention; therefore, findings are not reflective of physicians who chose not to review their data. As our recruitment was limited to a single site with an established QI program [12], future work should explore whether these findings are consistent across a range of primary care physicians in different settings and geographical locations. This type of future work is critical to identify strategies to promote intervention coherence and A&F uptake within the primary care community to effect change at a population level.

## Conclusions

As health systems continue to invest in initiatives that leverage routinely collected data to provide A&F, identifying how best to tailor feedback to recipient characteristics is likely to be a critical link to optimizing the benefit of A&F for population health. Our work suggests that a well-designed A&F intervention is necessary but not sufficient to successfully improve quality of care in the short term [29, 30]. Simply put, A&F initiatives—even those that are well designed—must include co-interventions to address recipient characteristics (i.e., beliefs and capabilities) and context to optimize impact. Co-interventions should provide support targeting the interpretation of longitudinal data, understanding common data sources and limitations, how aggregate data provides insights into individualized care, and identifying practice-level actions to improve quality. More broadly, undergraduate medical education and post-graduate training opportunities should embed curricula to ensure physicians are equipped with QI capabilities, with an emphasis on the skills required to engage meaningfully with QI initiatives.

## Supplementary Information

**Additional file 1. **Example feedback report.

**Additional file 2. **Interview Guide.

**Additional file 3. **Self-Reflection Guide.

**Additional file 4. **Self-Reflection Response.

## Data Availability

An aggregate summary of the data generated during this study is included in this published article. Individual data transcripts cannot be shared publicly due to confidentiality.
