# Supplementary material for: Unpacking the intention to action gap: a qualitative study understanding how physicians engage with audit and feedback
Source: Implement Sci. 2021 Feb 17;16:19. doi: 10.1186/s13012-021-01088-1 (PMC7891166; doi:10.1186/s13012-021-01088-1)
Supplement: Supplementary file 1 — Additional file 1. Example feedback report. [file 13012_2021_1088_MOESM1_ESM.pdf]

St. Michael's
Inspired Care. Inspiring Science.
Academic Family Health Team

Data for Practice Reflection

Dr. Example
Updated May 17, 2018

Your Patient Characteristics & Complexity

Age and Gender Distribution for Your Patients

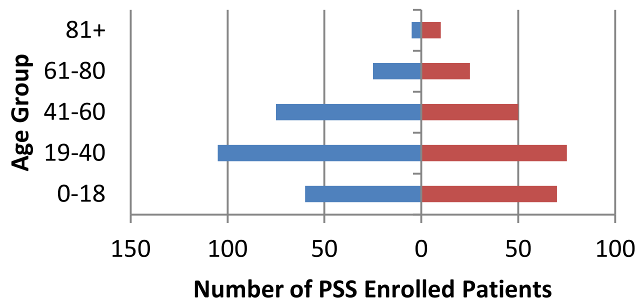

- 3 patients are coded as transgender or gender non-binary

Neighbourhood Income Quintile for Your Patients

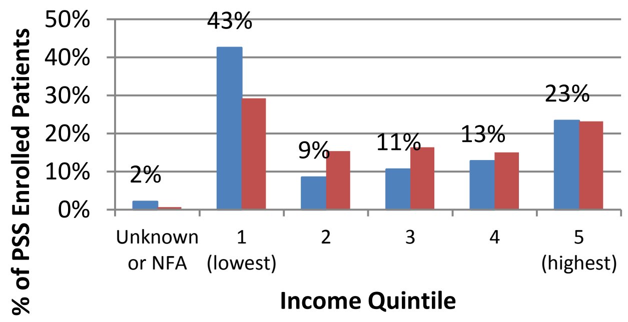

Data from PSS unless otherwise specified	Your Practice	Clinic Site	FHT
Diabetes	10% (50/500)	6%	7%
HIV	3% (15/500)	3%	3%
COPD	4% (20/500)	2%	2%
Developmental Delay	0.2% (1/500)	0.2%	0.2%
Schizophrenia	2% (10/500)	3%	3%
Bipolar	3% (15/500)	2%	1%
Addictions	2% (10/500)	2%	2%
Previous M.I. (Health Quality Ontario data)	4%	Not available	1% (Ontario)
SAMI Score (Health Quality Ontario data)	1.3	Not available	1.2
Registered with OHIP in the last 10 years, proxy for immigration (Health Quality Ontario data)	21%	Not available	14%
SMH medical service discharges in past year	11 discharges; 7 unique patients	207 discharges; 128 unique patients	1145 discharges; 790 unique patients

Your Practice Profile and Rostering

Your Practice		Clinic Median		FHT Median	
MOH Roster Size	Clinical FTE	MOH Roster Size	Clinical FTE	MOH Roster Size	Clinical FTE
510	0.5	564	0.6	573	0.6
MOH Roster Size/cFTE: 1020		MOH Roster Size/cFTE: 940		MOH Roster Size/cFTE: 955	

PSS Roster Statuses for Your Patients

Enrolled or postponed	Declined	Ineligible	Inappropriate	Not rostered, seen in the last year	FHO terminated, seen after termination date and in last year
500/520	0/520	5/520	5/520	5/520	5/520

The # of PSS enrolled/postponed patients may not match the # of MOH rostered patients. "Your Practice" refers to your enrolled/postponed patients in PSS, except for Health Quality Ontario data and cancer screening rates.

% of patients with:	Your Practice	Clinic Site	FHT
Email addresses on file	50% (250/500)	34%	39%
Health Equity survey completed	25% (125/500)	29%	36%

St. Michael's
Inspired Care.
Inspiring Science.

Academic Family Health Team

Data for Practice Reflection

Dr. Example
Updated May 17, 2018

Access and Continuity – Health Quality Ontario Data

	Your Practice	FHT	LHIN	Ontario (not adjusted)
Continuity: % of your patients' primary care visits made to you (includes visits to FHT and non-FHT MDs)	65%	55%	68%	69%
All emergency department visits (Adjusted rate per 1000 patients)	550.0	514.4	380.8	404.4
Urgent emergency department visits (CTAS 1-3) (Adjusted rate per 1000 patients)	370.0	327.8	247.2	263.0
Less urgent emergency department visits (CTAS 4-5) (Adjusted rate per 1000 patients)	190.0	177.9	132.0	140.0

Your Third Next Available Appointment

Third Next Available Appointment (TNA)

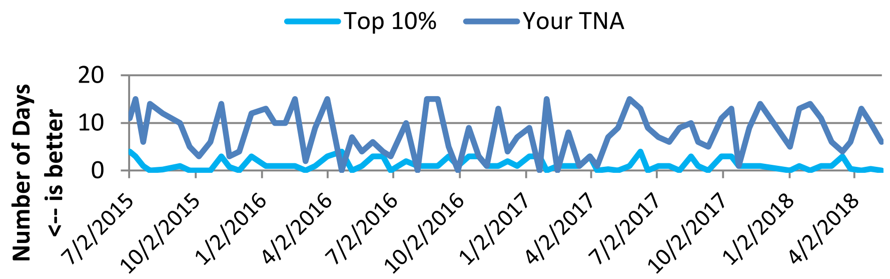

Over the past 6 months:

Your mean TNA	9 days
FHT 90th percentile	1 day

Prescribing for Your Rostered Patients

PSS Prescribing for Your Patients, Over Time

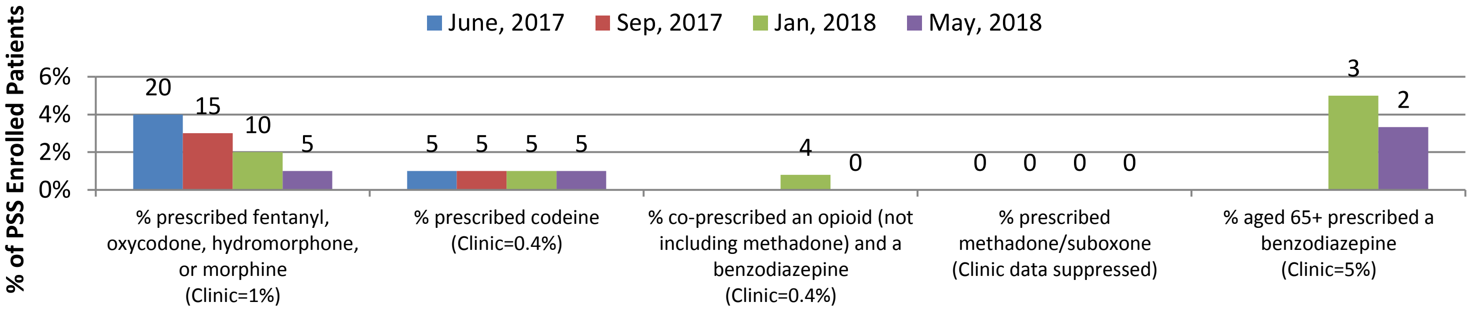

Health Quality Ontario Prescribing Data for Your Patients, Over Time

What percentage of my non-palliative care patients have been newly dispensed an opioid prescription (excluding opioid agonist therapy) within the last 6 months?

- As of March 31, 2017, 4.5% of my patients have been newly dispensed an opioid prescription. 12.5% of those opioids were prescribed by me and 87.5% were prescribed by other providers (e.g., other family physicians, dentists, surgeons).
- My group and LHIN percentages are 4.1% and 4.0%, respectively. The provincial percentage is 4.2%. **These percentages are for context only and do not represent a target.**

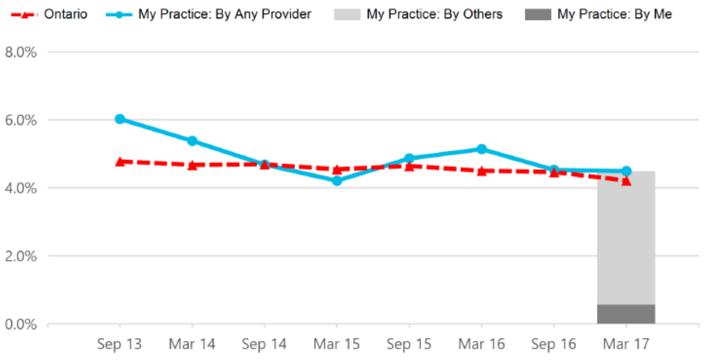

† Data suppressed as per ICES' privacy policy (e.g. number of patients between 1 to 5); N/A: Data not available; ¨ Please interpret with caution, denominator ≤ 30

What percentage of my non-palliative care patients have at least one high-dose opioid >90 mg MEQ daily within the last 6 months?

- As of March 31, 2017, † of my patients have a high-dose opioid >90 mg MEQ daily. † of those opioids were prescribed by me and † were prescribed by other providers (e.g., other family physicians, dentists, surgeons).
- My group and LHIN percentages are 1.0% and 0.7%, respectively. The provincial percentage is 0.9%. **These percentages are for context only and do not represent a target.**

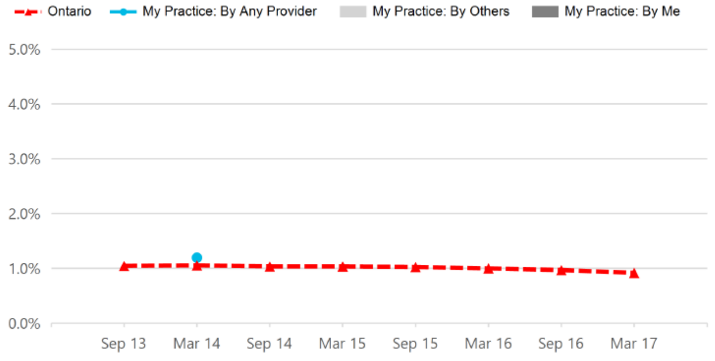

† Data suppressed as per ICES' privacy policy (e.g. number of patients between 1 to 5); N/A: Data not available; ¨ Please interpret with caution, denominator ≤ 30

St. Michael's
Inspired Care.
Inspiring Science.

Academic Family
Health Team

Data for Practice Reflection

Dr. Example
Updated May 17, 2018

Chronic Disease Prevention & Management for Your Rostered Patients

Cancer Screening Rates for Your Patients

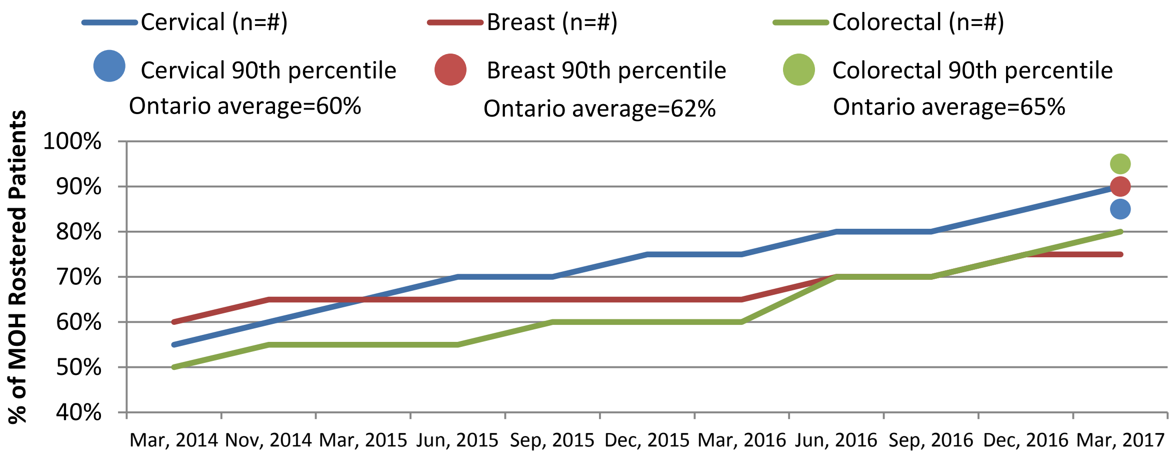

% screened or offered screening, June, 2017	
Cervical	95%
Breast	80%
Colorectal	85%

	Your Practice	Clinic Site	FHT
**Smoking Cessation**			
Patients who currently smoke who were advised to quit within the past year	33%<br>(20/60)	27%	22%
Patients **with COPD** who currently smoke who were advised to quit within the past year	67%<br>(10/15)	45%	36%
Patients **with diabetes** who currently smoke who were advised to quit within the past year	40%<br>(4/10)	47%	34%
**Immunizations**			
Patients aged 65+ with documentation of flu shot done within the past year	Sep, 2017: 50% (25/50)<br>May , 2018: 70% (35/50)	Sep, 2017: 33%<br>May, 2018: 35%	Sep, 2017: 33%<br>May, 2018: 29%
Patients aged 65+ with documentation of pneumovax	Sep, 2017: 60% (30/50)<br>May , 2018: 80% (40/50)	Sep, 2017: 50%<br>May, 2018: 54%	Sep, 2017: 56%<br>May, 2018: 57%
Patients aged 65-70 with documentation of Zostavax	May , 2018: 7% (1/15)	14%	16%
**Developmental Screening**			
Children aged 24-36 months with a Nipissing District Developmental Screen on file or A002 billed	Sep, 2017: 50% (5/10)<br>May , 2018: 90% (9/10)	Sep, 2017: 74%<br>May, 2018: 75%	Sep, 2017: 78%<br>May, 2018: 76%

Diabetes Care

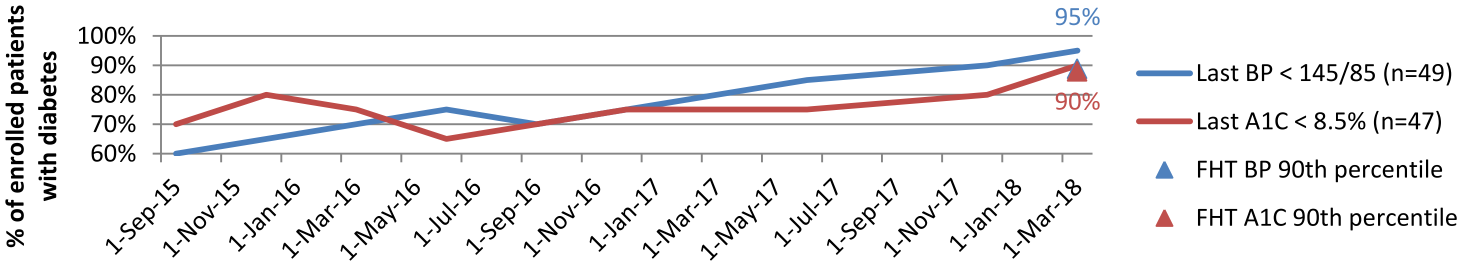

**Diabetes Care**	Your Practice	Clinic Site	FHT
Patients with Diabetes retained in care (BP and A1C, or endo visit within last 6 months)	Sep, 2017: 50% (25/50)<br>May , 2018: 80% (40/50)	Sep, 2017: 64%<br>May, 2018: 63%	Sep, 2017: 61%<br>May, 2018: 64%
Aged over 40 and currently prescribed a statin	Sep, 2017: 94% (45/48)<br>May , 2018: 94% (45/48)	Sep, 2017: 67%<br>May, 2018: 70%	Sep, 2017: 72%<br>May, 2018: 73%
Retinopathy screening (Health Quality Ontario data)	70%	Not available	68%

St. Michael's
Inspired Care.
Inspiring Science.

Academic Family Health Team

Data for Practice Reflection

Dr. Example
Updated May 17, 2018

Your Team's Patient-Reported Access and Continuity

Access to Care When Sick, Over Time

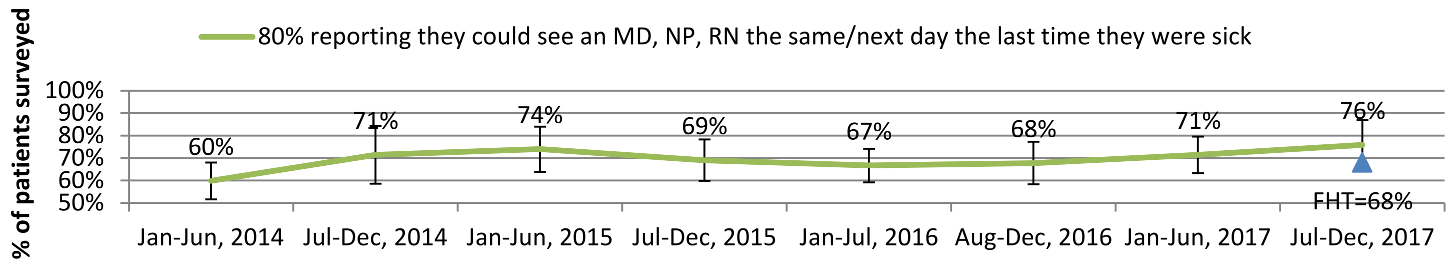

Same Day Answers to Medical Questions, Over Time

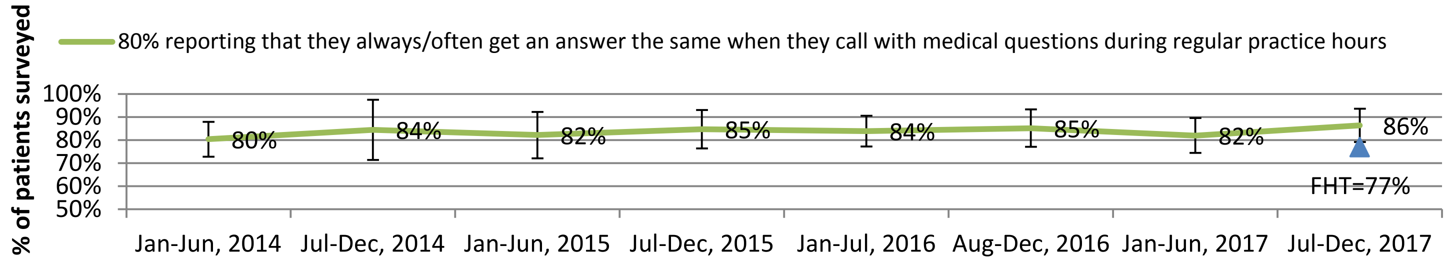

After-Hours Access, Over Time

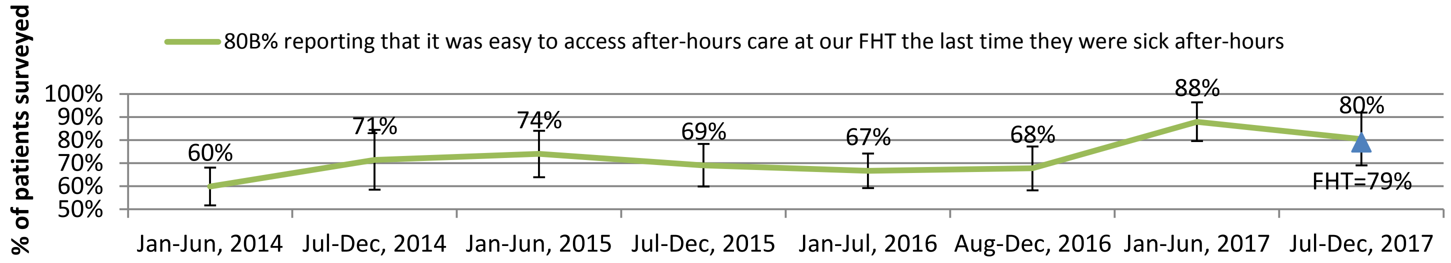

Satisfaction with Booked Appointments and Waiting Room Wait Times, Over Time

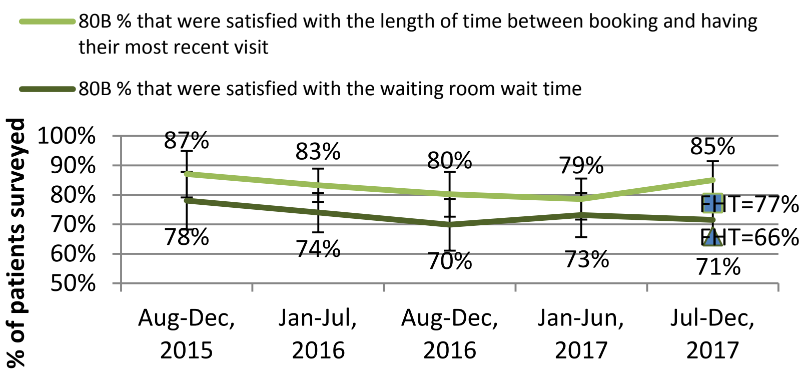

Continuity Over Time

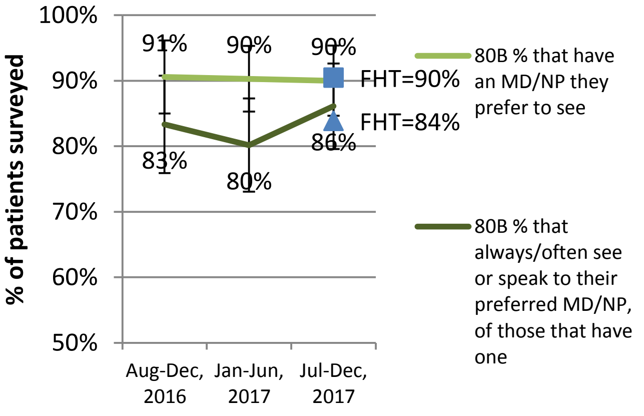

Your Patient Experience Survey Comments

<u>What do you like best about being a patient?</u>

"Quote"

"Quote"

"Quote"

"Quote"

"Quote"

"Quote"

St. Michael's
Inspired Care.
Inspiring Science.

Academic Family
Health Team

Data for Practice Reflection

Dr. Example
Updated May 17, 2018

Measure	Source	How Measure is Calculated
Age and gender	PSS search (May 1, 2018)	Age and gender as listed in PSS for your enrolled and postponed patients, not including patients coded as transgender (see below)
Patients coded as transgender	PSS search (May 1, 2018)	# of your rostered and postponed patients coded as identifying as transgender or gender non-binary
Neighbourhood income quintile	PSS search (May 1, 2018); 2006 census Postal Code Conversion File	# with no postal code in PSS, or postal code mapped to income quintile, as determined by 2006 census; divided by the number with no postal code in PSS or valid income quintile
# of patients with diabetes	PSS search (Apr 6, 2018)	# coded as having diabetes, divided by your # of enrolled and postponed patients
# of patients with HIV	PSS search (May 4, 2018)	# coded as having HIV, divided by your # of enrolled and postponed patients
# of patients with COPD	PSS search (May 1, 2018)	# coded as having COPD, divided by your # of enrolled and postponed patients
# of patients with developmental delay	PSS search (May 2, 2018)	# in the developmental delay cohort, divided by your # of enrolled and postponed patients
# of patients with schizophrenia	PSS search (Mar 31, 2018)	# of enrolled or postponed patients with a diagnostic code 295, or a service code Q021A within the past year; divided by your # of enrolled and postponed patients
# of patients with bipolar	PSS search (Mar 31, 2018)	# of enrolled or postponed patients with a diagnostic code 296, or a service code Q020A within the past year; divided by your # of enrolled and postponed patients
# of patients with addictions	PSS search (Mar 31, 2018)	# of enrolled or postponed patients with a diagnostic code 303 or 304; or a service code K682A, K683A, K684A, or K680A within the past year; divided by your # of enrolled and postponed patients
Previous M.I.	Health Quality Ontario's Primary Care Practice Report (Mar 31, 2017)	# of patients with a most responsible diagnosis of AMI based on the ICD-9 code 410 or ICD-10 code I21, divided by the # of MOH rostered patients as of March 31, 2016<br><br>See http://www.hqontario.ca/Quality-Improvement/Guides-Tools-and-Practice-Reports/Primary-Care for details
SAMI Score	Health Quality Ontario's Primary Care Practice Report (Mar 31, 2017)	See http://www.hqontario.ca/Quality-Improvement/Guides-Tools-and-Practice-Reports/Primary-Care for details
Registered with OHIP in the last 10 years	Health Quality Ontario's Primary Care Practice Report (Mar 31, 2017)	Your MOH rostered patients with a first registration in OHIP within the last 10 years excluding children <10<br><br>See http://www.hqontario.ca/Quality-Improvement/Guides-Tools-and-Practice-Reports/Primary-Care for details
SMH medical service discharges in past year	Transition to Home program (extracted apr 25, 2018)	# of discharges and unique patients where you are identified as the family physician
Roster Size, cFTE	Roster reports, and billing group<br>Extracted Apr, 2018	For clinic and FHT: median roster size is divided by median cFTE
PSS roster statuses	PSS search (May 1, 2018)	# of patients with your name listed as their doctor in PSS
% with email	PSS search (May 1, 2018)	# with email address on file or @declined, divided by the # of enrolled or postponed patients
% with Health Equity data	PSS search (May 4, 2018)	# with Health Equity survey on file, divided by the # of enrolled or postponed patients

St. Michael's
Inspired Care. Inspiring Science. | Academic Family Health Team

Data for Practice Reflection

Dr. Example
Updated May 17, 2018

Measure	Source	How Measure is Calculated
Continuity: % of your patients' primary care visits made to you	Health Quality Ontario's Primary Care Practice Report (Mar 31, 2017)	# of primary care visits to the physician in the past 2 years by patients rostered or virtually rostered to the physician, divided by total number of primary care visits in the system in the past 2 years by patients rostered or virtually rostered to the physician. Excludes children < 1 year of age.<br><br>See http://www.hqontario.ca/Quality-Improvement/Guides-Tools-and-Practice-Reports/Primary-Care for details
All emergency department visits	Health Quality Ontario's Primary Care Practice Report (Mar 31, 2017)	# of ED visits for conditions measured as CTAS level 1-5 in the previous year, divided by total number of patients in the previous year. Excludes visits with an inpatient admission, and children < 1 year of age. Adjusted for complexity.<br><br>See http://www.hqontario.ca/Quality-Improvement/Guides-Tools-and-Practice-Reports/Primary-Care for details
Urgent emergency department visits	Health Quality Ontario's Primary Care Practice Report (Mar 31, 2017)	# of ED visits for conditions measured as CTAS level 1-3 in the previous year, divided by total number of patients in the previous year. Excludes visits with an inpatient admission, CTAS 4-5, planned ED visits, and children < 1 year of age. Adjusted for complexity.<br><br>See http://www.hqontario.ca/Quality-Improvement/Guides-Tools-and-Practice-Reports/Primary-Care for details
Less urgent emergency department visits	Health Quality Ontario's Primary Care Practice Report (Mar 31, 2017)	# of ED visits for conditions measured as CTAS level 4-5 in the previous year, divided by total number of patients in the previous year. Excludes visits with an inpatient admission, CTAS 1-3, planned ED visits, and children < 1 year of age. Adjusted for complexity.<br><br>See http://www.hqontario.ca/Quality-Improvement/Guides-Tools-and-Practice-Reports/Primary-Care for details
TNA	PSS schedules (updated May 9, 2018)	# of days until your third next available appointment, counting pink slots if they are on the same day or the next day as the day the TNA is being collected
PSS prescribing for your patients, all indicators	PSS search (May 5-8, 2018)	# of patients with you listed as the doctor in PSS who have been prescribed these medications within the past 6 months and have the medication listed in their current medication list, divided by the # of enrolled or postponed patients
Newly-dispensed opioids	Health Quality Ontario's Primary Care Practice Report (Mar 31, 2017)	% of rostered non-palliative care patients newly dispensed an opioid (by you and by other providers) within a 6-month reporting period. Opioid agonist therapy (OAT), cough and antidiarrheal opioid medications not included.<br><br>See http://www.hqontario.ca/Quality-Improvement/Guides-Tools-and-Practice-Reports/Primary-Care for details
High-dose opioids	Health Quality Ontario's Primary Care Practice Report (Mar 31, 2017)	% of rostered non-palliative care patients with a high-dose (> 90 MEQ) opioid product(s) on at least one day within a 6-month reporting period. Opioid agonist therapy (OAT), cough and antidiarrheal opioid medications were not included in the opioid definition.<br><br>See http://www.hqontario.ca/Quality-Improvement/Guides-Tools-and-Practice-Reports/Primary-Care for details
Cancer screening	Cancer Care Ontario Screening Activity Report updated Mar 31, 2018; PSS search run Apr 24, 2018	% of eligible patients (ie. no surgery, not currently being treated for cancer, no exclusions as per Q codes or PSS reminder annotations) with a pap within 3 years; mammogram within 2 years; colonoscopy within 10 years, FOBT within 2 years, flex sig within 5 years<br><br>Informed discussion includes patients who have declined, or been informed, as documented in the PSS reminder annotations, within the numerator
Smoking cessation	PSS search (Apr 29, 2018)	# of enrolled or postponed patients whose risk factors or OMSC forms list them as a "current smoker"; and have been advised to quit within the last year as documented in OMSC forms, or by billing code E079A, K039A, or Q042A ; divided by the # of enrolled or postponed patients whose risk factors or OMSC forms list them as a "current smoker"

St. Michael's
Inspired Care. Inspiring Science.
Academic Family Health Team

Data for Practice Reflection

Dr. Example
Updated May 17, 2018

Measure	Source	How Measure is Calculated
65+ with flu shot done within past year	PSS search (Apr 10, 2018)	# of enrolled or postponed patients who have a documented flu shot in PSS within the past year and are aged 65+, divided by the # of enrolled or postponed patients aged 65+
65+ with pneumovax	PSS search (May 5, 2018)	# of enrolled or postponed patients who have a documented pneumovax in PSS and are aged 65+, divided by the # of enrolled or postponed patients aged 65+
Nipissing District Developmental Screening	PSS search (May 7, 2018)	# of enrolled or postponed patients aged 24-36 months who have a NDDS on file or A002 billed, divided by the # of enrolled or postponed patients aged 24-36 months
Diabetes outcomes	PSS search (Apr 6, 2018)	% of PSS rostered and postponed patients with their last systolic BP <145 and their last diastolic BP <85 (out of those with a BP on file); % of PSS rostered and postponed patients with their A1C <8.5% (out of those with an A1C on file)
Diabetes care indicators: retained in care	PSS search (Apr 6, 2018)	# of enrolled or postponed patients with diabetes who have either a) BP and A1C done within 6 months; or b) endo visit within 6 months; divided by # of enrolled/postponed patients with diabetes
Diabetes care indicators: aged over 40 and prescribed a statin	PSS search (Apr 6, 2018)	# of enrolled or postponed patients with diabetes aged over 40 and whose current medication list contains a statin; divided by the # of enrolled/postponed patients with diabetes aged over 40
Diabetes care indicators: retinopathy screening	Health Quality Ontario's Primary Care Practice Report (Mar 31, 2018)	# of MOH rostered patients with diabetes aged 40 years and older who have had at least 1 retinal exam with an ophthalmologist or optometrist in the past 24 months, divided by # of diabetic patients aged 40 years and older. Excludes women with gestational diabetes.<br><br>See http://www.hqontario.ca/Quality-Improvement/Guides-Tools-and-Practice-Reports/Primary-Care for details
Access to care when sick, over time	Patient Experience Survey (Dec, 2017)	% of surveyed patients who reported that they could see an MD, NP, or RN on the same or next day the last time they were sick and needed care, out of those who reported that they've had an urgent need in the past 12 months
Same day answers to medical questions, over time	Patient Experience Survey (Dec, 2017)	% of patients who reported that they always or often get an answer the same day when the call the clinic with medical questions during regular practice hours
After-hours access, over time	Patient Experience Survey (Dec, 2017)	% of patients who reported that the last time they were sick and needed care in the evening, weekend, or on a holiday, they could easily get care at our FHT within going to an outside "walk-in clinic" or the ED
Satisfaction with booked appointments	Patient Experience Survey (Dec, 2017)	% of patients who reported that thinking of the visit they just had, they would rate the wait time between making the appointment and having the appointment as excellent or very good
Satisfaction with waiting room wait time	Patient Experience Survey (Dec, 2017)	% of patients who reported that thinking of the visit they just had, they would rate the wait time in the waiting room as excellent or very good
Continuity	Patient Experience Survey (Dec, 2017)	% of patients who report having an MD or NP they prefer to see<br><br>Of those that have an MD or NP they prefer to see, the % of patients who report that they always or often see that MD or NP
