# Supplementary material for: Unpacking the intention to action gap: a qualitative study understanding how physicians engage with audit and feedback
Source: Implement Sci. 2021 Feb 17;16:19. doi: 10.1186/s13012-021-01088-1 (PMC7891166; doi:10.1186/s13012-021-01088-1)
Supplement: Supplementary file 2 — Additional file 2. Interview Guide. [file 13012_2021_1088_MOESM2_ESM.docx]

**Additional file 2 - Interview Guide**

*Note: Parts in italics not read to participant*

***Introduction***

1. What do you think about the data for practice reflection and the self-reflection guide you received?
   1. What insights did you glean from the data?

***D1: Coherence:*** *the ways people make sense of the work of implementing and integrating an intervention; to what extent does the feedback align with their way of thinking and what are their current beliefs*

1. *Can you describe how you view your practice and whether and how the data for practice reflection aligns with your practice?
   1. What was the impact of receiving your personalized data?

*Follow-up (if needed):* Did the data change the way you viewed your practice?

- 1. What role did the self-reflection guide have, if any?

*Follow-up (if needed):* Did the self-reflection guide influence the way you viewed your practice?

***D2: Cognitive Participation (Buy-in):*** *how they engage with it e.g. are they cognitively buying into data*

1. *How have you used the data for practice reflection in your clinical practice?
   1. Can you describe an example of how you used this data in your clinical practice?
2. What changes could be made to improve your ability to engage with your data?

*Probes: consider the indicators, data presentation, administrative and clinic factors (e.g., protected time for reviewing data)*

5. What strategies, if any, do you use to find the time and knowledge to reflect on the data amongst other competing demands?

***D3: Collective Action:*** *how they enact it; what are people doing about the feedback*

1. *Is there anything you have done or intend to do in response to the data?
   1. If yes, can you describe?

*Follow-up:* Is there something you want to do but couldn’t get around to doing? What circumstances prevented you from doing it?

- 1. If no, how could the feedback be improved to make it more useful to you?

1. Is there anything your team/colleagues have done or could do in response to the data?

***Priority Setting***

1. Do you prioritize areas for practice improvement? If so, how do you determine priority areas?
2. Did the data for practice reflection influence your priorities for professional development? If so, how? If not, could it be improved to better support you in your prioritization efforts?

***D4: Reflexive Monitoring:*** *appraise its effects*

1. *In your opinion, how important is it to monitor the impact of [the changes mentioned above] to your practice?

*Follow-up:* Do you have a plan for how to monitor the impact of the changes that you/your team made?

1. *How do you know or will you know that the changes you are making will have a positive effect?
   1. How can you be supported with this?
   2. What do you need to help you monitor the impact?

***Concluding Questions***

We’re just about near the end here. Thank you for answering all those questions so far. The last few questions focus on where we are at with this study and what has been rolled out.

1. Was the self-reflection guide a valuable resource for you?
   1. If yes, how did it help you process your practice data? If not, how could it be improved to become a better resource?
   2. Did the self-reflection guide influence:
      1. How you processed and interpreted your practice data?
      2. The insights you gleaned from the data?
      3. The changes you made (or plan to make) to your practice?
      4. Your professional development plans/actions?
2. How can we better support you to use the data for learning and improvement?
   1. What supports could you use to better understand and reflect on the data?
   2. What supports could you use to make improvements?
   3. What supports could you use for professional development?
3. Is there anything you would like to add that I may have left out?

Thank you for your time today.

*End recorder.*
