# Supplementary material for: Unpacking the intention to action gap: a qualitative study understanding how physicians engage with audit and feedback
Source: Implement Sci. 2021 Feb 17;16:19. doi: 10.1186/s13012-021-01088-1 (PMC7891166; doi:10.1186/s13012-021-01088-1)
Supplement: Supplementary file 3 — Additional file 3. Self-Reflection Guide. [file 13012_2021_1088_MOESM3_ESM.docx]

**Additional file 3 – Self-Reflection Guide**

**I. What do the data say?**

Please take a moment to reflect on your **clinic-level** data

1. Looking at the **clinic-level** data, in which areas does **your clinic** seem to be **faring well**?

Opioid Prescribing

- - Overall
  - Co-prescribing with benzodiazepines
  - Methadone/Suboxone
  - New starts
  - High-dose prescribing
- Prescribing: Aged 65+ prescribed a benzodiazepine

Preventive Care: Smoking Cessation

- - Overall
  - For patients with COPD
  - For patients with diabetes

Preventive Care: Immunizations

- - Flu shots for those age 65+
  - Pneumovax for those age 65+
- Preventive Care: Developmental Screening

Chronic Disease Management: Diabetes Care

- - % with A1C above target
  - % with BP above target
  - Retention in care
  - Statin prescribing
  - Retinopathy screening
- Patient-reported experience: Access to Care when Sick
- Patient-reported experience: Same Day Answers to Medical Questions
- Patient-reported experience: After-Hours Access
- Patient-reported experience: Satisfaction with Booked Appointments and Waiting Room Wait Times
- Patient-reported experience: Continuity

2. Still looking at the **clinic-level** data, in which areas does **your clinic** seem to be **improving over time?**

Preventive Care: Immunizations

- - Flu shots for those age 65+
  - Pneumovax for those age 65+
- Preventive Care: Developmental Screening

Chronic Disease Management: Diabetes Care

- - % with A1C above target
  - % with BP above target
  - Retention in care
  - Statin prescribing
- Patient-reported experience: Access to Care when Sick
- Patient-reported experience: Same Day Answers to Medical Questions
- Patient-reported experience: After-Hours Access
- Patient-reported experience: Satisfaction with Booked Appointments and Waiting Room Wait Times
- Patient-reported experience: Continuity

3. Thinking about your responses to the questions above, what areas would benefit from **further improvement effort** **for your clinic**?

Opioid Prescribing

- - Overall
  - Co-prescribing with benzodiazepines
  - Methadone/Suboxone
  - New starts
  - High-dose prescribing
- Prescribing: Aged 65+ prescribed a benzodiazepine

Preventive Care: Smoking Cessation

- - Overall
  - For patients with COPD
  - For patients with diabetes

Preventive Care: Immunizations

- - Flu shots for those age 65+
  - Pneumovax for those age 65+
- Preventive Care: Developmental Screening

Chronic Disease Management: Diabetes Care

- - % with A1C above target
  - % with BP above target
  - Retention in care
  - Statin prescribing
  - Retinopathy screening
- Patient-reported experience: Access to Care when Sick
- Patient-reported experience: Same Day Answers to Medical Questions
- Patient-reported experience: After-Hours Access
- Patient-reported experience: Satisfaction with Booked Appointments and Waiting Room Wait Times
- Patient-reported experience: Continuity

This section asked you about your **clinic-level** data. If you based your responses on interpretations of your personal practice or FHT-level data, please go back and review your responses and revise to be based on your **clinic-level** data.

Now take a moment to reflect on **your own practice** data

1. Looking at your **personal practice data**, in which areas do you seem to be **doing well**?

- Roster size
- Roster accuracy
- Access and Continuity: Continuity
- Access and Continuity: ED visit rates
- Third Next Available Appointment

Opioid prescribing

- - Overall
  - Co-prescribing with benzodiazepines
  - Methadone/Suboxone
  - New starts
  - High-dose prescribing
- Prescribing: Aged 65+ Prescribed a Benzodiazepine

Preventive Care: Cancer Screening

- - Cervical cancer screening
  - Breast cancer screening
  - Colorectal cancer screening

Preventive Care: Smoking Cessation

- - Overall
  - For patients with COPD
  - For patients with Diabetes

Preventive Care: Immunizations

- - Flu shots for those age 65+
  - Pneumovax for those age 65+
- Preventive Care: Developmental Screening

Chronic Disease Management: Diabetes Care

- - % with A1C above target
  - % with BP above target
  - Retention in care
  - Statin prescribing
  - Retinopathy screening

2. Continuing to reflect on your own **personal practice data**, in which areas do you seem to be **improving over time**?

- Third Next Available Appointment

Opioid prescribing

- - Overall
  - Co-prescribing with benzodiazepines
  - Methadone/Suboxone
  - New starts
  - High-dose prescribing
- Prescribing: Aged 65+ Prescribed a Benzodiazepine

Preventive Care: Cancer Screening

- - Cervical cancer screening
  - Breast cancer screening
  - Colorectal cancer screening

Preventive Care: Immunizations

- - Flu shots for those age 65+
  - Pneumovax for those age 65+
- Preventive Care: Developmental Screening

Chronic Disease Management: Diabetes Care

- - % with A1C above target
  - % with BP above target
  - Retention in care
  - Statin prescribing

3. Thinking about your responses to the two questions above, what areas do you think you may need **further improvement effort**?

- Roster size
- Roster accuracy
- Access and Continuity: Continuity
- Access and Continuity: ED visit rates
- Third Next Available Appointment

Opioid prescribing

- - Overall
  - Co-prescribing with benzodiazepines
  - Methadone/Suboxone
  - New starts
  - High-dose prescribing
- Prescribing: Aged 65+ Prescribed a Benzodiazepine

Preventive Care: Cancer Screening

- - Cervical cancer screening
  - Breast cancer screening
  - Colorectal cancer screening

Preventive Care: Smoking Cessation

- - Overall
  - For patients with COPD
  - For patients with Diabetes

Preventive Care: Immunizations

- - Flu shots for those age 65+
  - Pneumovax for those age 65+
- Preventive Care: Developmental Screening

Chronic Disease Management: Diabetes Care

- - % with A1C above target
  - % with BP above target
  - Retention in care
  - Statin prescribing
  - Retinopathy screening

4. What was the most surprising or interesting thing about your **own data**?

__________________________________________________________________________________________________________________________________________________________________________________________________________________________________________________

This section asked you about your **personal practice-level** data. If you based your responses on interpretations of your clinic-level or FHT-level data, please go back and review your responses and revise to be based on your **personal practice-level** data.

**II. Who might I talk to about the data?**

1. Thinking about an area where you seem to be achieving greater scores than others, what practice tips could you share with the group?

__________________________________________________________________________________________________________________________________________________________________________________________________________________________________________________

__________________________________________________________________________________________________________________________________________________________________________________________________________________________________________________

__________________________________________________________________________________________________________________________________________________________________________________________________________________________________________________

__________________________________________________________________________________________________________________________________________________________________________________________________________________________________________________

__________________________________________________________________________________________________________________________________________________________________________________________________________________________________________________

__________________________________________________________________________________________________________________________________________________________________________________________________________________________________________________

__________________________________________________________________________________________________________________________________________________________________________________________________________________________________________________

__________________________________________________________________________________________________________________________________________________________________________________________________________________________________________________

**III. How can I use the data?**

Now consider how you can use the date for learning and improvement.

1. Reflecting on the data, describe a goal for your own learning and professional development

2. Thinking about the next six months, what change(s) would you like to make in your practice?

3. What support(s) would enable you to make the practice change(s)?

4. Please provide any other reflections on your own data, clinic-wide data, or FHT-wide data

**IV. Making the data and feedback process more useful**

The following questions will help us evaluate the process of reflecting on your practice data and make it more useful to you.

I felt comfortable interpreting the data

Strongly disagree Disagree Neutral Agree Strongly agree

1 2 3 4 5

I learned something new from reviewing the data

Strongly disagree Disagree Neutral Agree Strongly agree

1 2 3 4 5

I will think about making changes to the way that I practice as a result of reviewing my data

Strongly disagree Disagree Neutral Agree Strongly agree

1 2 3 4 5

I will make a change in how I practice as a result of reviewing my data

Strongly disagree Disagree Neutral Agree Strongly agree

1 2 3 4 5

I would be comfortable discussing my practice data with a trusted colleague

Strongly disagree Disagree Neutral Agree Strongly agree

1 2 3 4 5

I plan to discuss my practice data with a trusted colleague

Strongly disagree Disagree Neutral Agree Strongly agree

1 2 3 4 5

I would be comfortable discussing my practice data with a group of physician colleagues

Strongly disagree Disagree Neutral Agree Strongly agree

1 2 3 4 5

I plan to discuss my practice data with a group of physician colleagues?

Strongly disagree Disagree Neutral Agree Strongly agree

1 2 3 4 5

I would be comfortable discussing my practice data with a group of physician and non-physician colleagues

Strongly disagree Disagree Neutral Agree Strongly agree

1 2 3 4 5

I plan to discuss my practice data with a group of physician and non-physician colleagues

Strongly disagree Disagree Neutral Agree Strongly agree

1 2 3 4 5

1. Please comment on what can be done to make this data and feedback process more useful for you. Please provide examples where possible.

__________________________________________________________________________________________________________________________________________________________________________________________________________________________________________________

2. Please tell us if there is any other data or information that you would find helpful to receive

_____________________________________________________________________________________________________________________________________________________________________________________________________________________________________________________________________________
