# Supplementary material for: Unpacking the intention to action gap: a qualitative study understanding how physicians engage with audit and feedback
Source: Implement Sci. 2021 Feb 17;16:19. doi: 10.1186/s13012-021-01088-1 (PMC7891166; doi:10.1186/s13012-021-01088-1)
Supplement: Supplementary file 4 — Additional file 4. Self-Reflection Response. [file 13012_2021_1088_MOESM4_ESM.docx]

**Additional file 4– Self-Reflection Responses**

| **Response Category** | **Example Response** |
| --- | --- |
| **Question: Please comment on what can be done to make this data and feedback process more useful for you.** | |
| Improve accuracy of data | *“Improve accuracy of data - I think there are several discrepancies between my actual screening rates (having recently done my preventative care bonuses) and what is outlined here.”* |
| Improve ability to interpret | *Could summarize top 3 areas where I am doing well, and top 3 areas to improve.* |
| Desire for support/discussion | *I would like to review my data with a colleague that I trust who has a similar roster size/patient population.* |
| General feedback to improve report | *Simplify and standardize terms used.* |
| General reflection | *I like that this is provided both electronically and hard copy. I had thought that I would prefer the electronic copy but I actually think the hard copy may be easier to flip through.* |
| No plan to act | *It is very hard to find the time to incorporate this activity with so many other competing priorities, which is why I don't plan on discussing this data with others / taking this reflection further.* |
| No comment | n/a |
| **Question: Reflecting on the data, describe a goal for your own learning and professional development.** | |
| Identified area for practice change | *Increased smoking cessation counselling.* |
| Improve knowledge | *I would like to read up on the new diabetes guidelines.* |
| Improve documentation | *For the purpose of cooperation with performance metrics I would like to improve my rates of documentation of smoking cessation discussions and reduce my [access indicator].* |
| Increase awareness | *Increased awareness of diabetes patients, their blood pressure, and whether they are on statins.* |
| Unclear how to improve | *How can I continue to decrease emergency room visit rates in my patients? It seems like [they] are well aware of after hour/on call services.* |
